# The long non-coding RNA lncMYOZ2 mediates an AHCY/MYOZ2 axis to promote adipogenic differentiation in porcine preadipocytes

**DOI:** 10.1186/s12864-022-08923-9

**Published:** 2022-10-11

**Authors:** Yang Yang, Yiqi Wu, Mengting Ji, Xiaoyin Rong, Yanwei Zhang, Shuai Yang, Chang Lu, Chunbo Cai, Pengfei Gao, Xiaohong Guo, Bugao Li, Guoqing Cao

**Affiliations:** College of Animal Science, Shanxi Agricultural University, Taigu, 030801 China

**Keywords:** lncRNA, lncMYOZ2, Porcine preadipocytes, Adipogenesis, AHCY, MYOZ2

## Abstract

Long non-coding RNAs (lncRNAs) play a vital role in regulating adipogenesis. However, the associated regulatory mechanisms have yet to be described in detail in pig. In this study, we demonstrate a critical role for lncMYOZ2 in adipogenesis from porcine preadipocytes. Specifically, lncMYOZ2 was more abundant in the adipose tissue of Mashen (fat-type) pigs than for Large White (lean-type) pigs, and knockdown of this lncRNA significantly inhibited the differentiation of porcine preadipocytes into adipocytes. Mechanistically, we used RNA pull-down and RIP assays to establish that lncMYOZ2 interacts with adenosylhomocysteinase (AHCY). Moreover, lncMYOZ2 knockdown increased promoter methylation of the target gene *MYOZ2* and lowered its expression. Finally, we describe a positive regulatory role for MYOZ2 in adipogenesis. Collectively, these findings establish lncMYOZ2 as an important epigenetic regulator of adipogenesis via the aforementioned AHCY/MYOZ2 pathway, and provide insights into the role of lncRNAs in porcine adipose development.

**Supplementary Information:**

The online version contains supplementary material available at 10.1186/s12864-022-08923-9.

## Introduction

Adipose tissue is essential for animals. It acts as the main energy storage organ and is central to the control of whole-body energy, directing endocrine control of fuel homeostasis by secreting a variety of adipokines [1, 2]. Adipose tissue is mainly composed of mature adipocytes, which are gradually differentiated from preadipocytes that form part of the stromal vascular fraction (SVF) [3]. Over recent years, a complex transcriptional and epigenetic cascade for regulation of adipogenesis has been identified [4, 5], but its exact functional mechanisms have yet to be fully clarified.

Long non-coding RNA (lncRNA) is generally defined as ncRNA molecules that does not encode proteins and is greater than 200 nucleotides (nt). Through the growing application of functional genomics research and transcriptome sequencing technology, some lncRNAs have been found to influence development and differentiation during chromatin modification, transcription, and post-transcriptional processing [6, 7]. Thus, lncRNAs could be central players in the integration of transcriptional and epigenetic factors during regulation of adipogenesis [8]. It is also important to note that most lncRNAs have obvious spatio-temporal expression specificity during tissue development. This varies across species, perhaps explaining their low sequence conservation and differing expression profiles [9]. In pig, the lncRNA PU.1 AS promotes adipogenesis from preadipocytes through formation of a sense–antisense RNA duplex with PU.1 mRNA [10]. Another lncRNA, IMFlnc1, acts as a competing endogenous RNA (ceRNA) to competitively bind miR-199a-5p, thereby upregulating expression of its target gene *CAV1* to promote intramuscular fat deposition [11]. Thus, delineation of lncRNA function in regulation of porcine adipogenesis is necessary to design more effective therapies.

Chinese indigenous Mashen pigs are a traditional fatty breed with higher fat content and intramuscular fat content than lean breeds [12, 13]. Studies have shown that expression of the key adipogenic regulators Zfp423, PPARγ, and CEBPα is significantly increased in the muscle tissue of Mashen pigs, indicating a strong capacity for adipogenesis [14]. To investigate the genetic regulatory mechanisms underlying these differences in adipogenesis between breeds, we previously compared the transcriptome of the longissimus dorsi between Mashen and Large White pigs [15]. We identified another regulatory lncRNA, lncMYOZ2, which was significantly more highly expressed in Mashen pigs, and its expression level was also positively correlated with that of its target gene, *MYOZ2*. In this study, the functional role of lncMYOZ2 in regulating adipogenesis was explored further, and provide a theoretical basis for in-depth study of the underlying molecular mechanisms.

## Materials and methods

### Animals and sample collection

Experimental animals were provided by the Datong Pig Breeding Farm (Shanxi, China). Three 90-day-old male Mashen pigs and Large White pigs were selected for slaughter. The biceps femoris, psoas, subcutaneous fat, spleen, liver, intestine, and stomach were collected from each animal and stored at − 80 °C.

### Porcine preadipocyte culture and adipogenic differentiation

A seven-day-old male Mashen pig was used for isolation of porcine preadipocytes based on previously established methods [16]. Subcutaneous adipose tissue (SAT) was digested using 0.1% type-I solution (Sigma) for 2 h at 37 °C, followed by centrifugation at 1000×*g* for 8 min. Afterwards, the resulting mixture was filtered through 100 μm and 40 μm mesh filters and centrifuged for another 8 min at 1000×*g* to obtain the porcine preadipocytes. After resuscitation, cells were cultured in DMEM (Gibco, USA) containing 10% fetal bovine serum (FBS; Gibco, USA) and 1% penicillin/streptomycin. For adipogenic differentiation, cells were treated with 1 μM dexamethasone (DEX), 0.5 mM 3-isobutyl-1-methylxanthine (IBMX), 10 mg/mL insulin, and 100 μM indomethacin for 2 days. The cells were then transferred to medium supplemented with 10% FBS and 10 mg/mL insulin for 2 days. Subsequently, they were maintained in 10% FBS for another 4 days.

### RNA interference and overexpression

To inhibit expression of lncMYOZ2, a customized siRNA preparation was used. This reagent contains a mixture of three synthetic siRNAs designed to target the intronic region of lncMYOZ2, with sequences (5′–3′) AGATGAACAACTGCTCTAA, AAGCTATGGTAACTAATCC, and GGAATGAAGCAATCTGACAG. A single siRNA was used to target *MYOZ2* (GACAAAGAAGATCTGACAA). The cDNA for lncMYOZ2 was obtained by gene synthesis (GeneCreate Inc., China) with flanking 5′-BamHI and 3′-XhoI sites and cloned into pCMV-N-FLAG using these restriction sites. For transfection, porcine preadipocytes were inoculated into 12-well plates and transfected with siRNA or plasmids using Lipofectamine 2000 (Invitrogen, Carlsbad, CA, USA), following the manufacturer’s instructions.

### RNA isolation and qPCR

Total RNA was extracted using the Trizol reagent (Life Technologies, USA) and transcribed into cDNA using a PrimeScript first strand cDNA synthesis kit (Takara Bio, Japan). Quantitation of mRNA levels by qPCR was performed on a real-time PCR system using SYBR Premix Ex Taq II (Takara Bio, Japan). The mean of the triplicate cycle thresholds (Ct) of the target gene was normalized to the mean of the triplicate Ct of the reference (18 s RNA) gene, using the 2^−ΔΔCt^ method, to yield relative gene expression levels. Primer sequences are listed in Supplementary Fig. S1.

### Cytoplasmic and nuclear fractionation

Cytoplasmic and nuclear fractions were isolated using the PARIS Kit (Thermo Fisher Scientific, USA) according to the manufacturer’s instructions. The RNA was extracted and reverse-transcribed into cDNA, then nuclear and cytoplasmic expression of lncMYZO2 were detected by qPCR. The positive controls were GAPDH (cytoplasmic) and U6 (nuclear).

### Bioinformatics analysis

Sequence analysis and positional information comparison of lncMYZO2 were performed using the NCBI (https://www.ncbi.nlm.nih.gov/) and Ensembl (http://asia.ensembl.org/index.html) websites. The CPC tool (http://cpc.cbi.pku.edu.cn/) was used to predict the protein-coding properties of lncMYOZ2.

### Western blot

Cells were extracted using a protein lysis buffer supplemented with protease inhibitor cocktail (Boster, China). Protein concentration was determined using a BCA kit (Boster, China). Then, samples were loaded at 20–30 μg/lane and separated on a 10% precast SDS–PAGE gel, followed by transfer onto PVDF membrane (Millipore, USA). After blocking with 5% skimmed milk powder (Sigma–Aldrich, USA) for 3 h, the membrane was incubated overnight at 4 °C with the appropriate primary antibody: against PPARγ (A0270, Abclonal, China), β-actin (AC038), FABP4 (A0232), CEBP-α (A0904), MYOZ2 (A6468), or AHCY (10,757, Proteintech, China). Then, IRDye® 800CW rabbit secondary antibodies (926-32,211, LI-COR, USA) were used to detect the primary antibodies, and the membrane was imaged using a far-infrared light scanning system (LI-COR, USA).

### Oil red O (ORO) staining

Eight days after adipogenic induction, cells were washed twice with phosphate buffered saline (PBS) and fixed in 4% paraformaldehyde for 30 min at room temperature. For staining, the cells were washed again with PBS and stained with freshly diluted ORO (Solarbio, China) for 15 min. After an additional wash with PBS, the cells were then imaged using a microscope (Leica, Germany). For quantitation of lipids, the dye retained in the cells (after washing) was eluted into isopropanol and the OD value at 510 nm was measured using a microplate reader (BioTEK, USA).

### RNA pull-down and RNA immunoprecipitation (RIP) assay

The sense and antisense strands of lncMYOZ2 were used as template for an in vitro transcription kit (GeneCreate, China) to obtain the target RNA. Following in vitro transcription, the biotin-labeled probes were incubated using a Pierce RNA 3′-End Desthiobiotinylation Kit (Thermo Fisher Scientific, USA). The biotin-labeled sequence were incubated with streptavidin coupled dynabeads (Invitrogen, USA) for 1 h at RT, and then with the lysate of porcine preadipocyte overnight at 4 °C, after which were separated by elution using 1D-SDS–PAGE and silver staining. Differentially expressed range in candidate region betwween two samples (sense and antisense) were then analyzed by mass spectrometry to assess the protein composition, which these proteins were then subjected to GO and KEGG enrichment analysis [17–19].

The RIP assay was performed using a RIP RNA-Binding Protein Immunoprecipitation Kit (Millipore, Bedford, MA, USA), in accordance with the manufacturer’s instructions. Briefly, cell lysates were incubated with magnetic beads conjugated with negative control (IgG) or anti-AHCY antibody. The immunoprecipitated RNAs were then extracted and analyzed by qPCR to confirm the enrichment of binding targets.

### DNA methylation

Genomic DNA was extracted from porcine preadipocytes using the phenol/chloroform extraction method, and bisulfite conversion was carried out with the EZ DNA Methylation Kit (ZYMO, USA) as per the manufacturer’s instructions. The promoter sequence of the pig *MYOZ2* gene was obtained from the NCBI website, and the MethPrimer (http://www.urogene.org/methprimer/) website was used to predict the methylation sites of *MYOZ2*. Thus, bisulfite-sequencing PCR (BSP) primers (F: GTTGGTGTTTTATTGTATGGTTGATT; R: ACAATCCCACTCCTAAACATCTATC) were designed and synthesized. The bisulfite-treated DNA was then amplified by PCR followed by cloning and sequencing. The sequencing results were analyzed using QUMA (http://quma.cdb.riken.jp/). The results are presented using a black solid circle to indicate that a site is methylated; a white circle to indicate that a site is unmethylated; and “×” to indicate a vacancy.

### Statistical analysis

All experiments were carried out with three biological replicates, and statistical analyses were performed using SPSS 22.0. Comparisons between two samples were made using the Student’s t-test, where *P* < 0.05 (*) and *P* < 0.01 (**) indicate statistically significant differences. Comparisons between three or more samples were performed using one-way analysis of variance (ANOVA), and Duncan’s method was used for multiple comparisons. Different capital letters indicate *P* < 0.05 (*), where the difference is statistically significant.

## Results

### Characteristics of pig lncMYOZ2

**Fig. 1:**
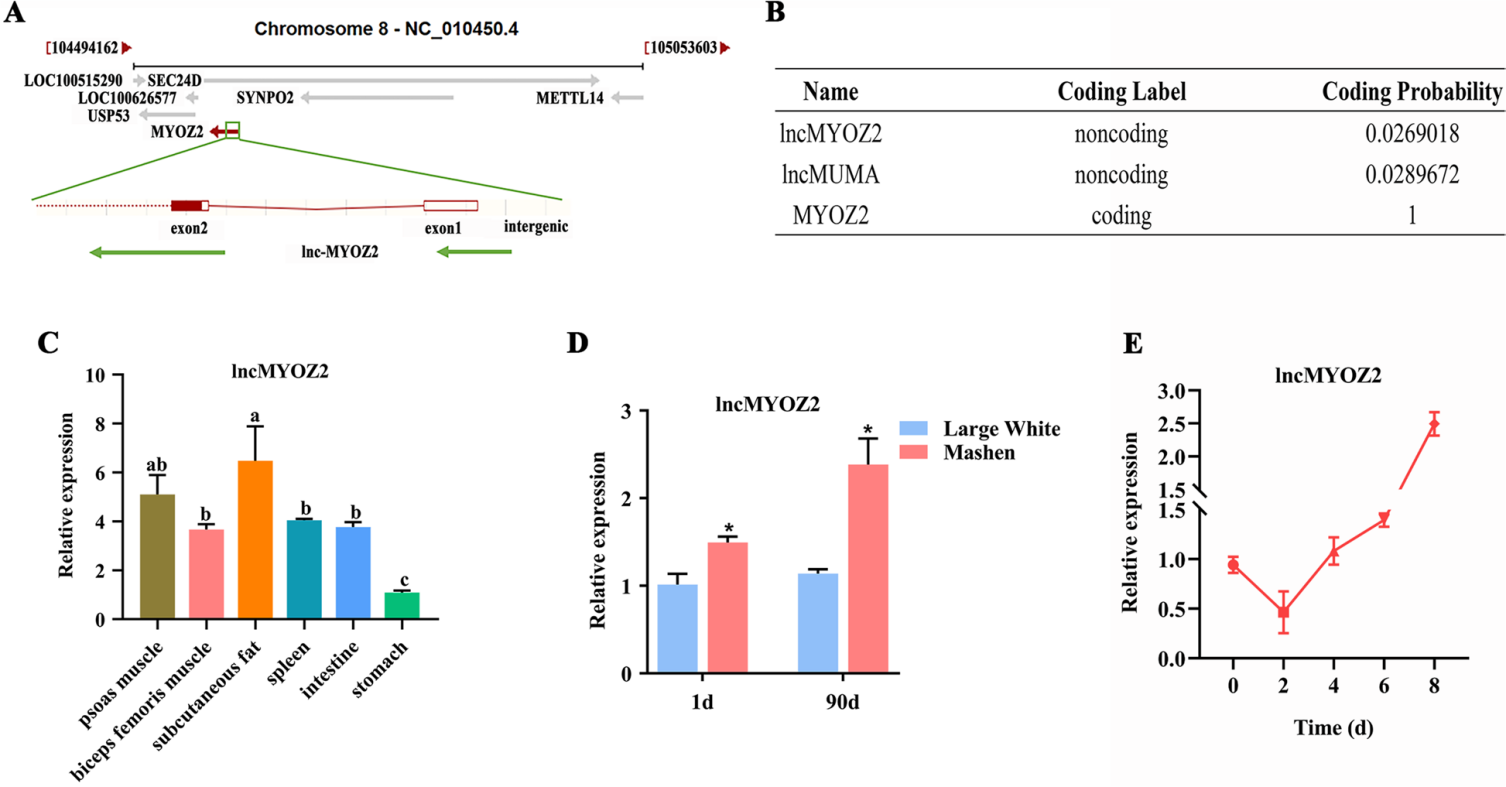
Identification of pig lncMYOZ2 characteristics. **A** Genomic localization of lncMYOZ2. **B** Coding potential of lncMYOZ2. **C** Expression of lncMYOZ2 in different tissues of Mashen pig was measured by quantitative PCR (qPCR). **D** Expression of lncMYOZ2 in subcutaneous adipose tissue of Mashen (fat-type) and Large White (lean-type) pigs. **E** Time course of lncMYOZ2 expression during adipogenic differentiation of porcine preadipocytes. Differentiation into adipocytes was induced using growth medium (DMEM with 10% FBS) supplemented with 0.5 mM IBMX, 1 uM DEX, 10 mg/mL insulin, and 100 μM indomethacin. Data are shown as mean ± standard error of the mean (SEM). Different letters show the significant expression of lncMYOZ2 in the different tissues of pig (*P* < 0.05). * (*P* < 0.05) indicates a statistically significant difference compared to control

LncMYOZ2 was transcribed from the sense strand of pig *MYOZ2* on chromosome 8. The transcript length was 709 nt, and aligning it against the genomic locus on UCSC, a overlap with intergenic, intron 1, intron 2, exons 1 and 2 of *MYOZ2* was identified (Fig. 1A). To exclude a protein-coding function for lncMYOZ2, its sequence was inserted into the CPC program for analysis, along with lncMUMA and *MYOZ2*. This confirmed that lncMYOZ2 has no protein-coding probability or potential (Fig. 1B).

Subsequently, we analyzed five tissue types from Mashen (fat-type) pigs and found the highest expression of lncMYOZ2 in muscle and adipose tissue (Fig. 1C). We also compared lncMYOZ2 expression levels in the adipose tissue of the two pig breeds, and found significantly higher expression in Mashen pigs than in Large White (lean-type) pigs (Fig. 1D). Furthermore, porcine preadipocytes were collected at various time points (0, 2, 4, 6 and 8 d) after adipogenic induction to research temporal changes. Expression of lncMYOZ2 was initially downregulated at 2 d post-induction, but it then increased gradually over the course of adipogenesis (Fig. 1E). Together, these results suggest that lncMYOZ2 may influence the function of adipose tissue in pigs.

### lncMYOZ2 accelerates adipogenesis of porcine preadipocytes

**Fig. 2:**
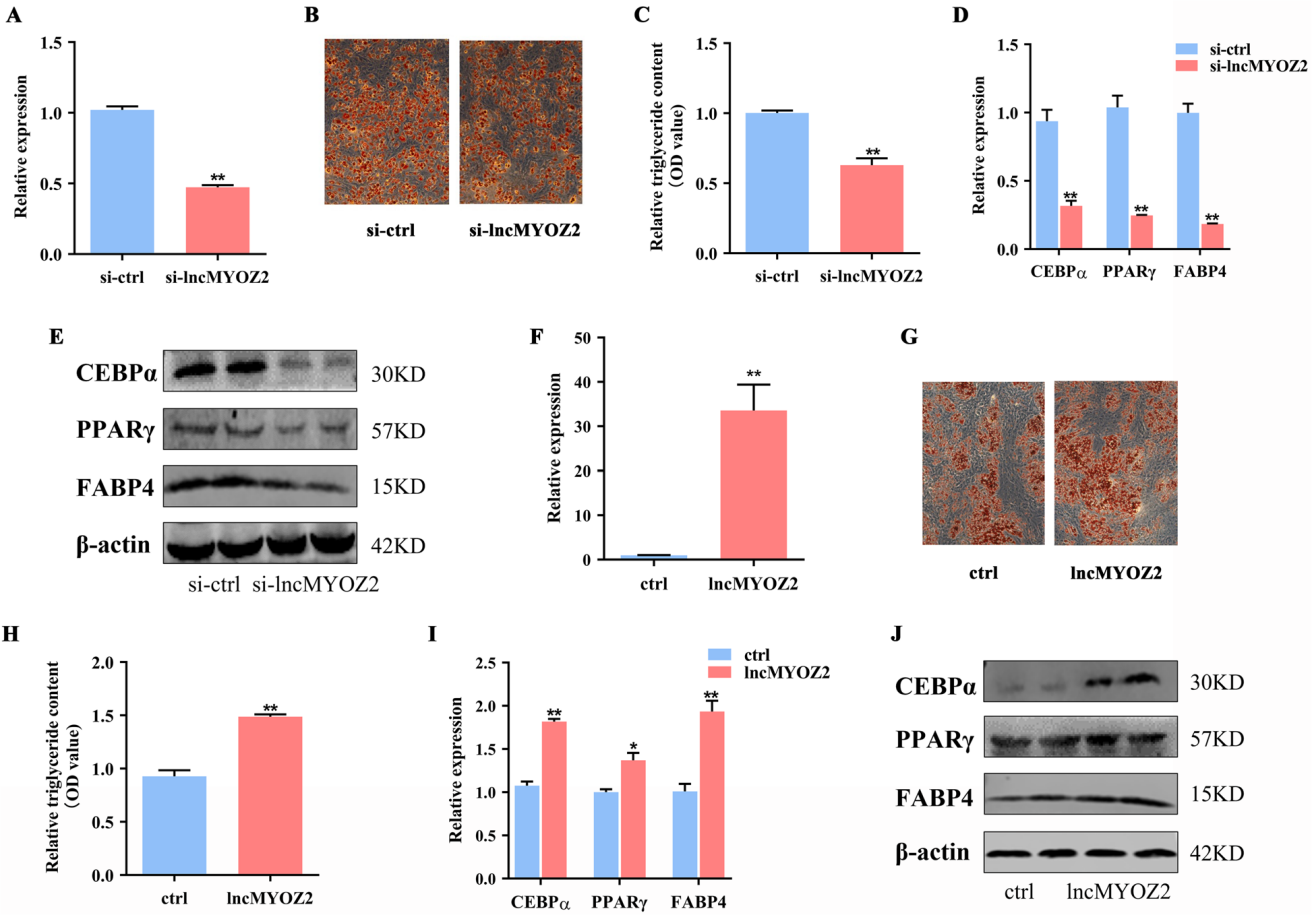
Effects of lncMYOZ2 knockdown on adipocyte differentiation. **A** Expression of lncMYOZ2 in porcine preadipocytes treated with siRNA mix for 36 h. **B**, **C** Adipogenic phenotypes of lncMYOZ2-knockdown or control porcine preadipocytes, after induction of adipogenic differentiation, as assessed by ORO staining (**B**) and triglyceride accumulation (**C**). The mRNA (**D**) and protein (**E**) levels of CEBPα，PPARγ and FABP4 at day 8 post-induction of adipocyte differentiation, as detected by qRT-PCR and Western blot. **F** The mRNA levels of lncMYOZ2-overexpression in porcine preadipocytes. **G**, **H** The adipogenic phenotypes of porcine preadipocytes transfected with lncMYOZ2-pCMV-N-FLAG or control after adipogenic induction for 8 days were assessed by ORO staining and triglyceride accumulation. **I**, **J** qPCR and Western blot analysis of CEBPα，PPARγ and FABP4 at day 8. Data are shown as mean ± SEM. * (*P* < 0.05) and ** (*P* < 0.01) indicate significant differences compared to control

To further evaluate the role of lncMYOZ2 in adipogenesis, we transfected porcine preadipocytes with a mixture of three siRNAs targeting lncMYOZ2 (Fig. 2A). Eight days after adipogenic induction, ORO staining of these lncMYOZ2-knockdown preadipocytes revealed drastically diminished lipid accumulation (Fig. 2B), along with reduced triglyceride content (Fig. 2C). Furthermore, mRNA levels of the adipocyte markers CEBPα, PPARγ, and FABP4 were reduced in lncMYOZ2-knockdown cells at the same time point (Fig. 2D). The protein levels of all these markers were also reduced in the treated cells (Fig. 2E). In addition, we observed that overexpression of lncMYOZ2 was able to accelerate adipogenesis from porcine preadipocytes (Fig. 2F–J). Taken together, these results indicate that lncMYOZ2 is a positive regulator on adipogenesis that enhances the rate of cellular differentiation into adipocytes.

### lncMYOZ2 interacts with the adenosylhomocysteinase (AHCY) protein

**Fig. 3:**
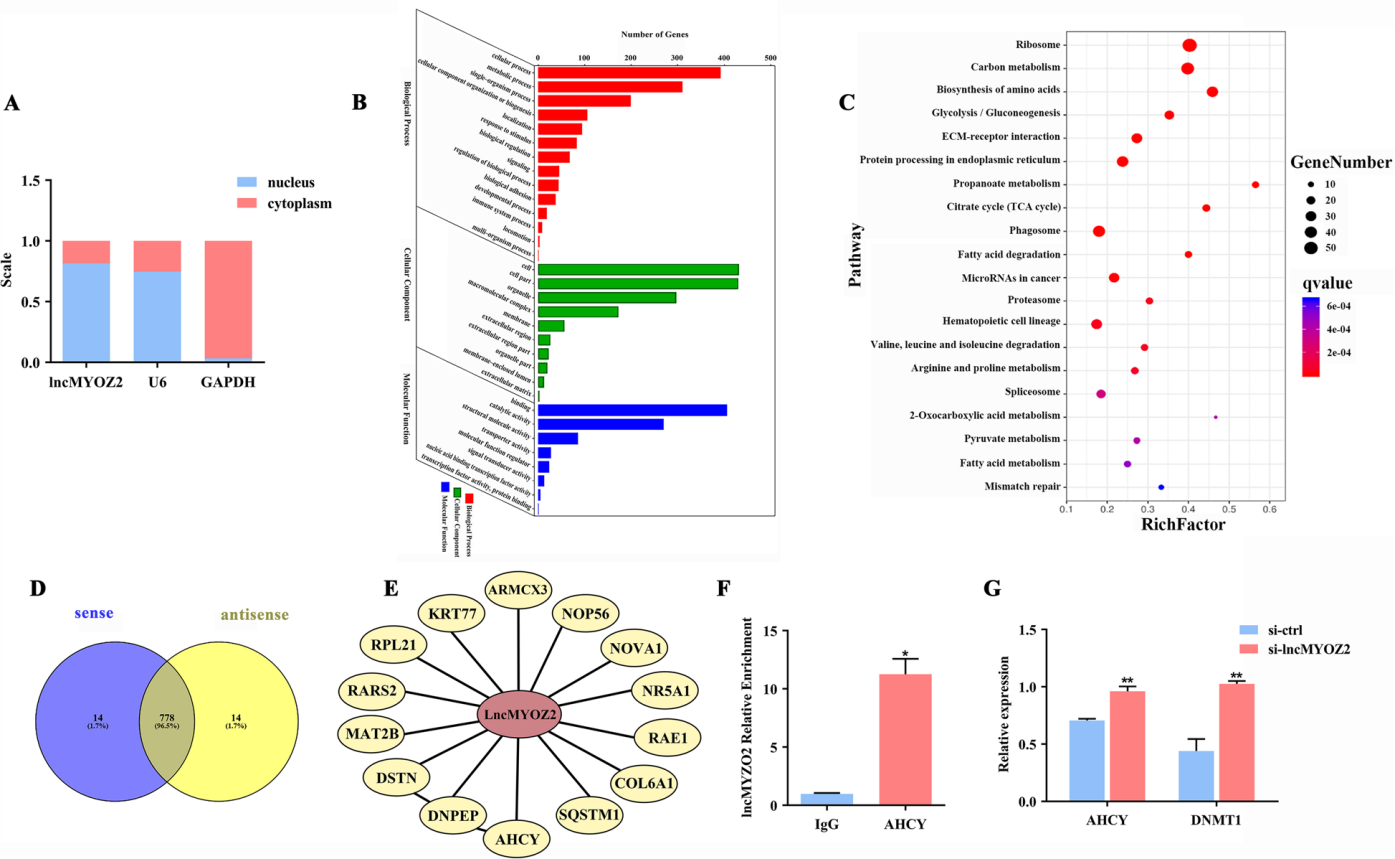
Functional screening of lncMYOZ2-interacting proteins. **A** Subcellular distribution of lncMYOZ2 in porcine preadipocytes. **B**, **C** GO and KEGG enrichment analysis showing the pathways related to lncMYOZ2. **D** Overlap of pulled-down proteins between sense and antisense transcripts. **E** The 14 proteins found distinctively in the interactome of the sense lncMYOZ2 transcript. **F** AHCY-RIP in porcine preadipocytes followed by qRT-PCR to detect endogenous association between AHCY and lncMYOZ2, IgG was served as the control. **G** mRNA levels of *AHCY* and *DNMT1* after lncMYOZ2 interference. Data are shown as mean ± SEM. * (*P* < 0.05) and ** (*P* < 0.01) indicate significant differences compared to control

To identify the potential mechanisms associated with these regulatory effects of lncMYOZ2, we first determined its subcellular distribution in porcine preadipocytes, and found that the majority of transcripts were located in the nucleus (Fig. 3A). Then, we prepared biotin-labeled sense or antisense lncMYOZ2 by in vitro transcription (Supplementary Fig. S1A), and co-incubated both samples with cell lysate of porcine preadipocytes in an RNA pull-down assay. Analysis by mass spectrometry (MS) revealed total 806 lncMYOZ2-associated proteins in the candidate region (Supplementary Fig. S1B, Table S2).

We subjected these proteins to GO and KEGG enrichment analysis (Supplementary Tables S3, S4), which showed that lncMYOZ2 was significantly related to the functions of RNA binding, catalytic activity, ribosome, carbon metabolism, and fatty acid-related metabolic pathways (Fig. 3B, C). Among the lncMYOZ2-associated proteins, 14 showed distinct interactions with the sense transcript (Fig. 3D, E). In addition, RIP-qPCR was used to validate binding of lncMYOZ2 with AHCY (Fig. 3F). Interestingly, we also found that the mRNA level of *AHCY* was enhanced with lncMYOZ2 knockdown, along with its downstream gene, *DNMT1* (Fig. 3G), suggesting that lncMYOZ2 functions sequentially through modulation of AHCY/DNMT1.

### lncMYOZ2 influences the DNA methylation level of MYOZ2

**Fig. 4:**
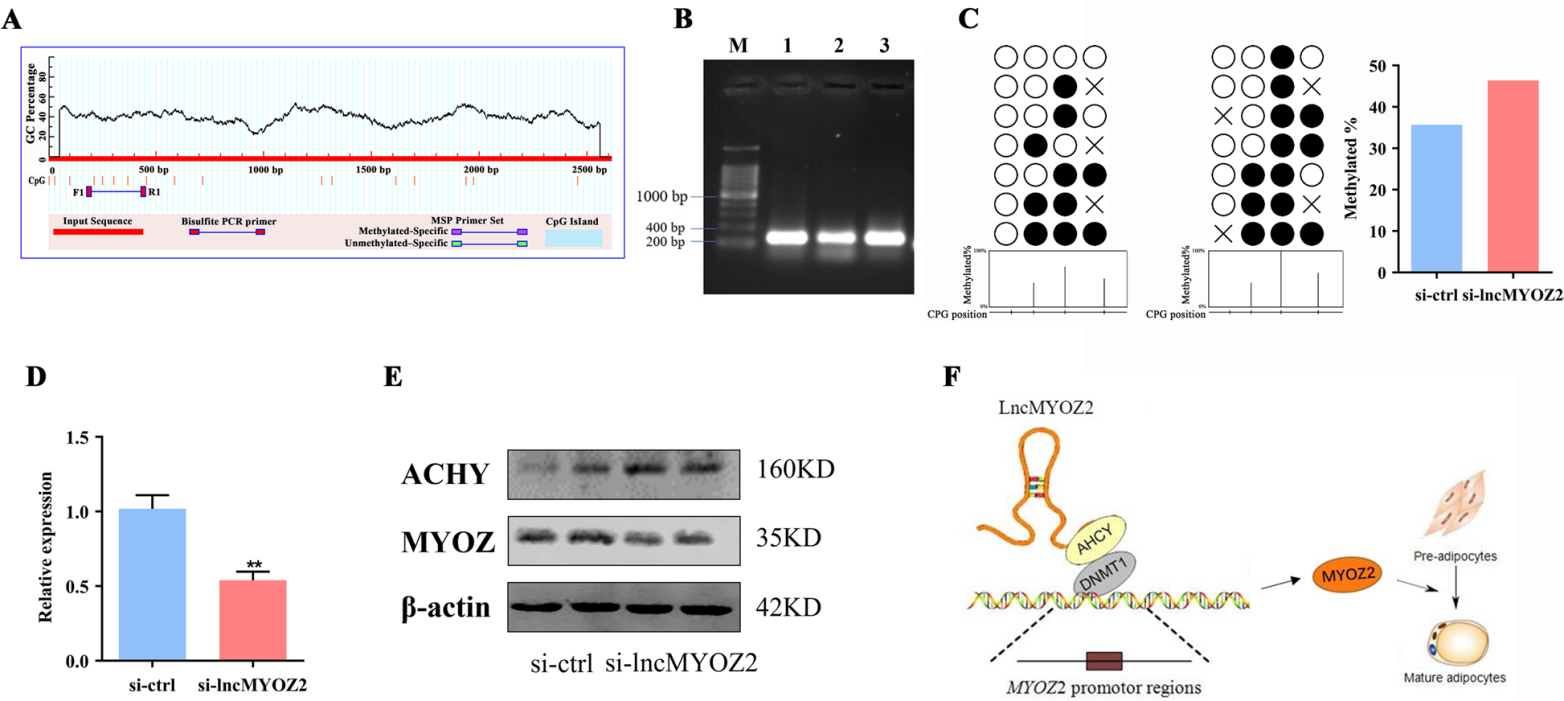
Influence of lncMYOZ2 on *MYOZ2* promoter methylation. **A** Prediction of DNA methylation sites in the promoter region of the *MYOZ2* gene. **B** Amplification of CpG sites in promoter region of *MYOZ2* gene; M: DL2000 DNA ladder. **C** Changes in *MYOZ2* promoter methylation pattern after interference with lncMYOZ2. **D** *MYOZ2* mRNA levels after lncMYOZ2 knockdown. **E** AHCY and MYOZ2 protein levels after lncMYOZ2 knockdown. **F** A proposed model of lncMYOZ2-dependent adipogenesis. Data are shown as mean ± SEM. * (*P* < 0.05) and ** (*P* < 0.01) indicate significant differences compared to control

The preceding results suggest that lncMYOZ2 downregulates DNMT1 expression. The remaining question was whether it could also influence DNA methylation levels of the target gene, *MYOZ2*. We used MethPrimer to predict DNA methylation sites in the *MYOZ2* promoter, and BSP primers were designed to examine the level of DNA methylation (Fig. 4A). Briefly, DNA was extracted from preadipocytes in the negative control (NC) and knockdown groups. The samples were then treated with bisulfite and PCR-amplified using the BSP primers, followed by Sanger sequencing (Fig. 4B).

Seven distinct promoter methylation patterns were identified for *MYOZ2*, in both the NC and knockdown groups. Among these, the NC group showed total vacancy at four loci (Fig. 4C), with statistical analysis also revealing that DNA methylation in the NC group was, in general, lower than that in the knockdown group (Fig. 4C). Furthermore, we found that knockdown of lncMYOZ2 inhibited expression of MYOZ2 (Fig. 4D, E). Together, these results suggest that lncMYOZ2 knockdown suppresses MYOZ2 expression by elevating DNA methylation of the gene promoter.

Collectively, our findings indicate that lncMYOZ2 facilitates adipogenesis of porcine preadipocytes in an AHCY/DNMT1-dependent manner to balance expression of MYZO2. Based on this, we propose a model for lncMYOZ2 regulation of adipogenesis (Fig. 4F).

### MYOZ2 promotes porcine preadipocyte differentiation

**Fig. 5:**
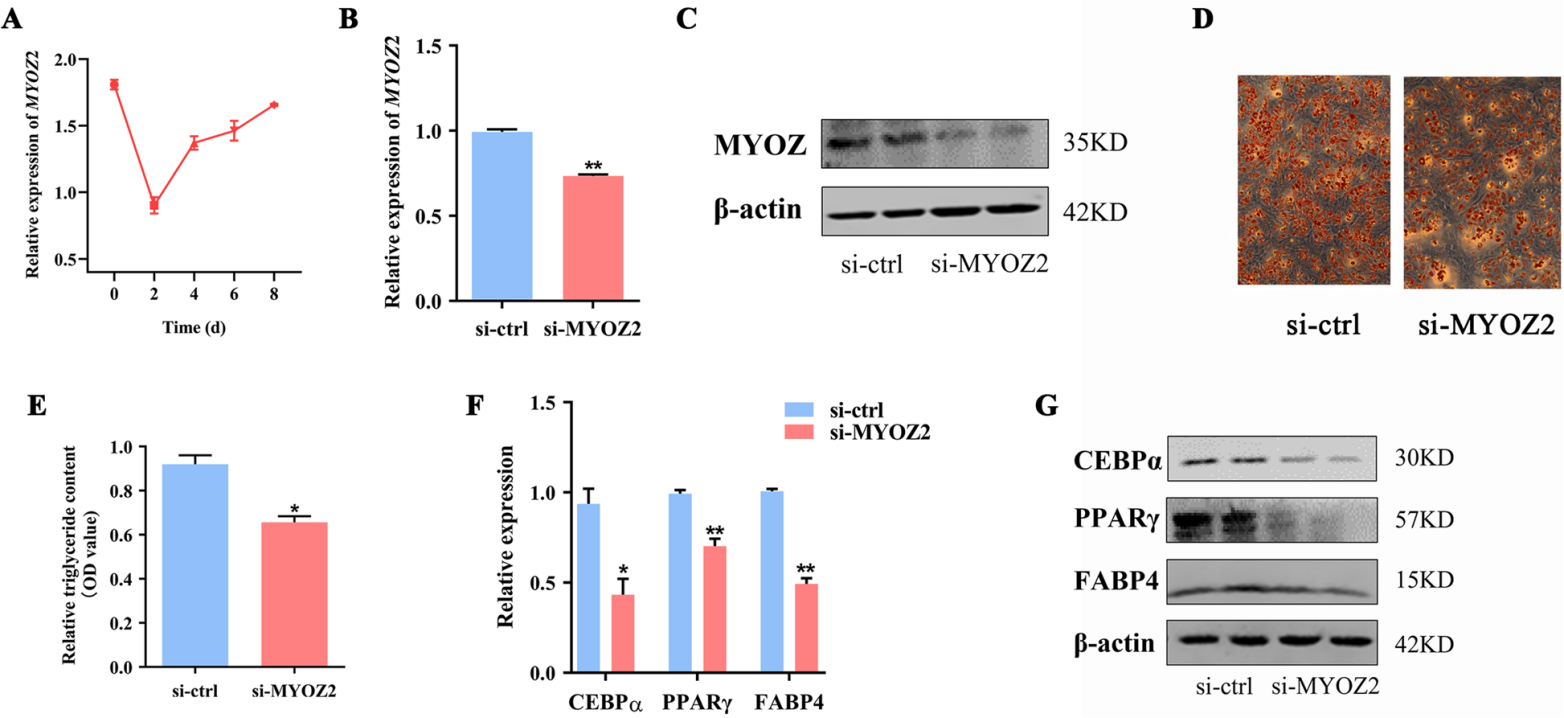
MYOZ2 accelerates adipogenic differentiation of porcine preadipocytes. **A** Time course expression profile of *MYOZ2* in porcine preadipocytes after induction of differentiation. mRNA (**B**) and Protein (**C**) expression of MYOZ2 in porcine preadipocytes treated with siRNA for 36 h. ORO staining (**D**) and triglyceride accumulation (**E**) showing adipogenic phenotypes of porcine preadipocytes with or without *MYOZ2* knockdown. **F** mRNA levels of *CEBPα*，*PPARγ* and *FABP4* at day 8 post-induction, as detected by qRT-PCR. **G** Protein levels of CEBPα，PPARγ and FABP4 at day 8, measured by Western blot. Data are shown as mean ± SEM. * (*P* < 0.05) and ** (*P* < 0.01) indicate significant differences compared to control

Given the apparent role of lncMYOZ2 in regulation of adipogenesis, we further evaluated whether its target gene, MYOZ2, functions in adipogenic differentiation. Expression of *MYOZ2* was downregulated 2 d after adipogenic induction but then increased gradually during the course of adipogenesis, mirroring the pattern seen for lncMYOZ2 (Figs. 1E, 5A). Next, the mRNA and protein levels of MYOZ2 were successfully downregulated by siRNA transfection of porcine preadipocytes (Fig. 5B, C). We found a drastic decrease in lipid accumulation in these *MYOZ2* knockdown cells 8 d after induction of adipogenesis (Fig. 5D, E). Additionally, the adipocyte markers CEBPα, PPARγ, and FABP4 were also reduced in MYOZ2 knockdown cells, at both the mRNA (Fig. 5F) and protein (Fig. 5G) levels. Overall, our findings establish an essential role for *MYOZ2* in regulating adipogenesis in pigs.

## Discussion

Obesity and its associated metabolic diseases are unabatedly growing in prevalence worldwide [20]. Thus, understanding the mechanisms of adipocyte differentiation could lead to promising new strategies for the treatment of obesity-related diseases. Historically, lncRNAs were known as “noise” generated by the transcription process, with no biological function. However, as the depth of research has increased, accumulating evidence has established that lncRNAs actually play vital roles in epigenetic regulation, cell proliferation, and cell differentiation [21]. As such, a number of lncRNAs regulating adipocyte differentiation have now been identified [22], although the relevant functional mechanisms in pig have not yet been fully elucidated.

In the current study, we have characterized the role of lncMYOZ2 in adipogenesis, finding that its expression is higher in fat-type (as opposed to lean-type) pigs. Moreover, gain-of-function and loss-of-function experiments have revealed that lncMYOZ2 acts as a positive regulator of porcine adipocytes differentiation, which sheds new light on the role of this epigenetic factor in adipogenesis in pig.

Other studies have shown that the function of lncRNAs is closely related to their subcellular localization. For example, lncRNAs distributed in the nucleus participate in epigenetic or transcriptional regulation of target genes by binding key proteins [23, 24]. In contrast, cytoplasmic lncRNAs can interact with RNA-binding proteins or competitively bind with miRNAs to regulate the stability and translation of mRNA [25, 26]. To identify potential mechanisms by which lncMYOZ2 might regulate adipogenesis, we firstly observed a predominantly nuclear distribution of its transcripts. Then, analysis of its interactome by RNA pull-down assays found 14 proteins that specifically bind the sense strand of lncMYOZ2. Among them, SFPQ, MAT2B, SQSTM1 and DSTN have been shown to be involved in the regulation of adipogenic differentiation [27–30], which suggested that lncMYOZ2 mainly regulates adipogenesis by processes involving these key proteins.

One of these lncMYOZ2 interactors, adenosylhomocysteinase (AHCY), acts as an adenosine homocysteine hydrolase and plays an important role in DNA methylation [31]. It is the only enzyme that can hydrolyze *S*-adenosylhomocysteine (SAH), a by-product of DNA methylation that acts as a feedback inhibitor of DNA methyltransferases, including DNMT1. Furthermore, it was reported that AHCY binds to DNMT1 to enhance its activity during DNA replication [32]. Since we found that AHCY also binds to lncMYOZ2, we further explored the specific role of lncMYOZ2 in DNA methylation. Expression of AHCY and DNMT1 was upregulated after lncMYOZ2 silencing, accompanied by enhanced DNA methylation of the *MYOZ2* promoter, and eventually, reduced MYOZ2 expression. Thus, lncMYOZ2 acts as an important epigenetic regulator coordinating the action of AHCY/DNMT1 to control expression of MYOZ2.

This protein, MYOZ2 (myozenin-2/calsarcin 1), was first reported to act as a link between calcineurin and muscle contraction elements [33]. It can form a feedback pathway with calcineurin to regulate the conversion of muscle fiber types, and increased expression of MYOZ2 decreases the activity of calcineurin and the number of slow muscle fibers. Conversely, lowered calcineurin activity can inhibit the expression of NFAT and MEF2, which in turn downregulates MYOZ2 to rescue the activity of calcineurin and maintain the number of slow muscle fibers [33, 34].

However, whether MYOZ2 is also involved in the regulation of adipogenesis deserves further attention. It was previously shown that expression of lncMYOZ2 is positively correlated with its source gene MYOZ2 [35], and in this work, we established *MYZO2* as a target gene of lncMYOZ2. After adipogenic induction, expression of lncMYOZ2 and MYZO2 showed the same trend, albeit with a relatively increase after the initial decrease. Furthermore, MYOZ2 knockdown also drastically diminished lipid accumulation in differentiated porcine preadipocytes. These findings are of great significance for understanding the mechanisms of lncMYOZ2 in influencing adipogenesis mediated by MYOZ2. They also suggest that continuing to elucidate the molecular pathways of MYOZ2 regulation of adipogenesis should be a focus of future work.

## Conclusions

We have identified a pivotal positive role of lncMYOZ2 in determining the differentiation of porcine preadipocytes into adipocytes. This function is mediated, at least in part, through regulation of AHCY/DNMT1 to modify *MYOZ2* promoter methylation and gene expression. Understanding the role of lncMYOZ2 in regulating adipogenesis should provide new insight into the mechanisms by which porcine preadipocytes undergo fate determination, and could form the molecular basis of new approaches to reduce the burden of obesity and related metabolic diseases.

## Supplementary Information

**Additional file 1.**

**Additional file 2.**

**Additional file 3.**

**Additional file 4.**

**Additional file 5.**

**Additional file 6.**

## Data Availability

The data sets supporting the conclusions of this article are included within the article and its additional files. The data sets used and/or analyzed during the current study are available from the corresponding author upon reasonable request.
