# Supplementary material for: The long non-coding RNA lncMYOZ2 mediates an AHCY/MYOZ2 axis to promote adipogenic differentiation in porcine preadipocytes
Source: BMC Genomics. 2022 Oct 11;23:700. doi: 10.1186/s12864-022-08923-9 (PMC9552422; doi:10.1186/s12864-022-08923-9)

Fig2E. CEBPα





Fig2E. FABP4


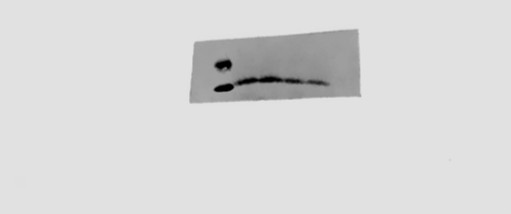


fig2E-actin


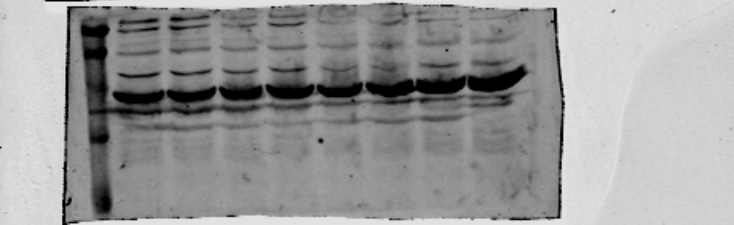


fig2E-PPARr


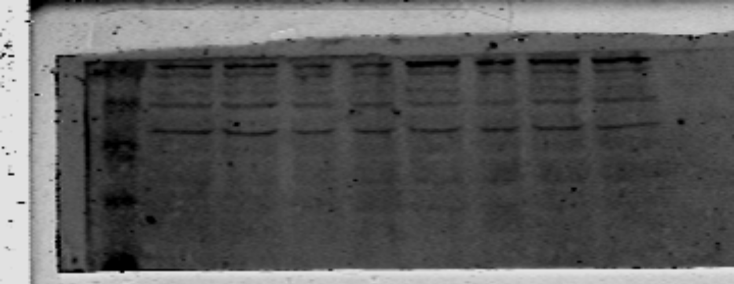


Fig2J. CEBPα


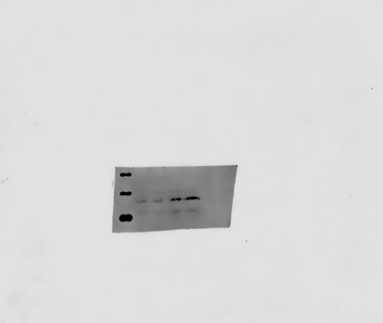


Fig2J. FABP4


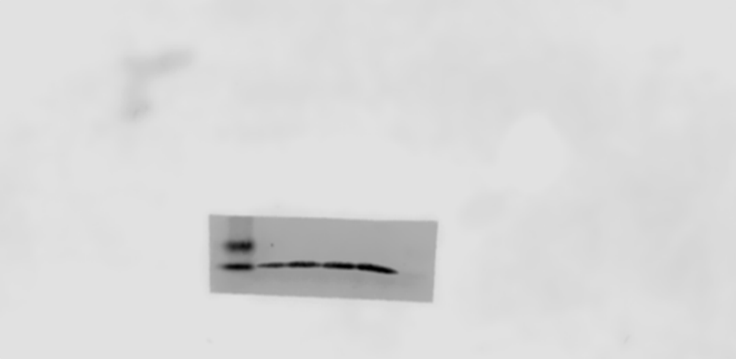


Fig2J. PPARγ





Fig2J. β-actin


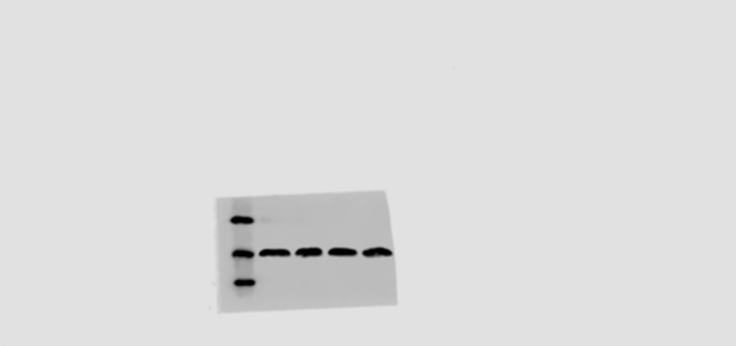


fig4b


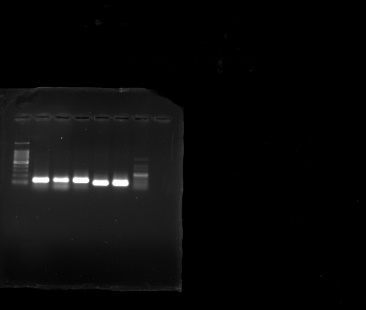


Fig4E. AHCY





Fig4E. MYOZ2


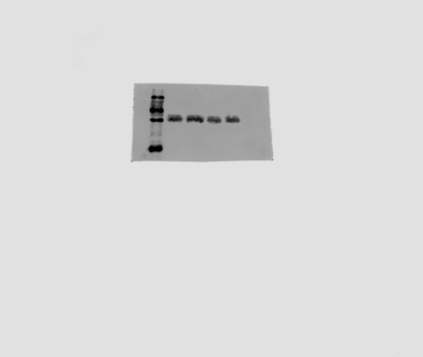


Fig4E. β-actin


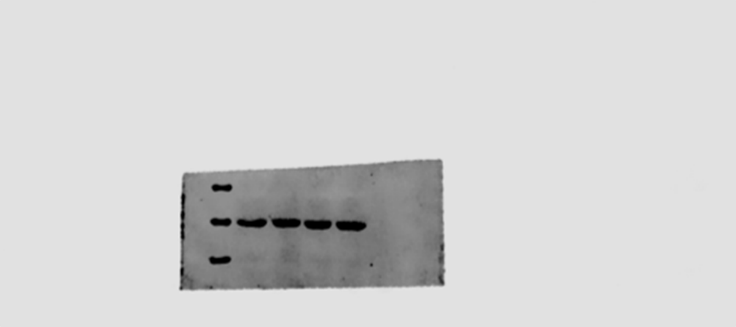


Fig5C. MYOZ2





Fig5C. β-actin


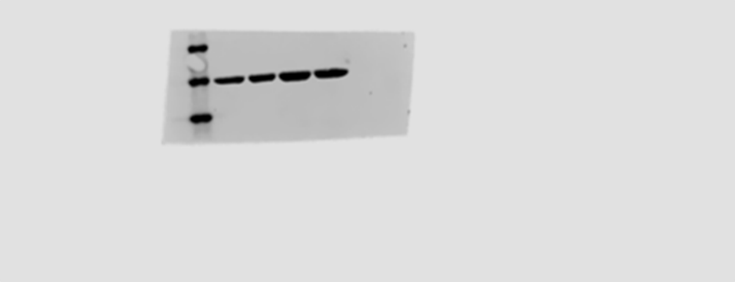


Fig5G. CEBPα





Fig5G. FABP4


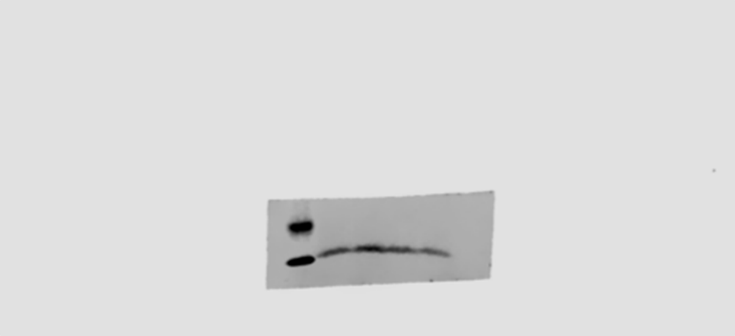


Fig5G. PPARγ





Fig5G. β-actin


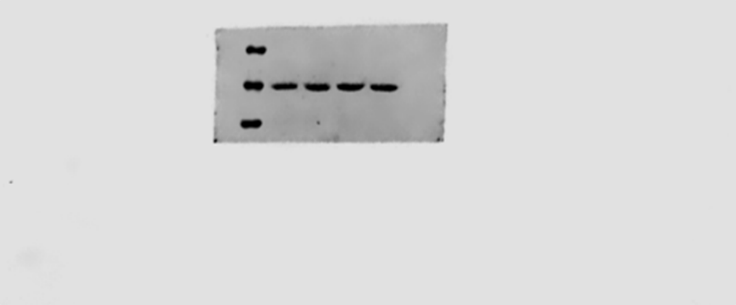


figS1A


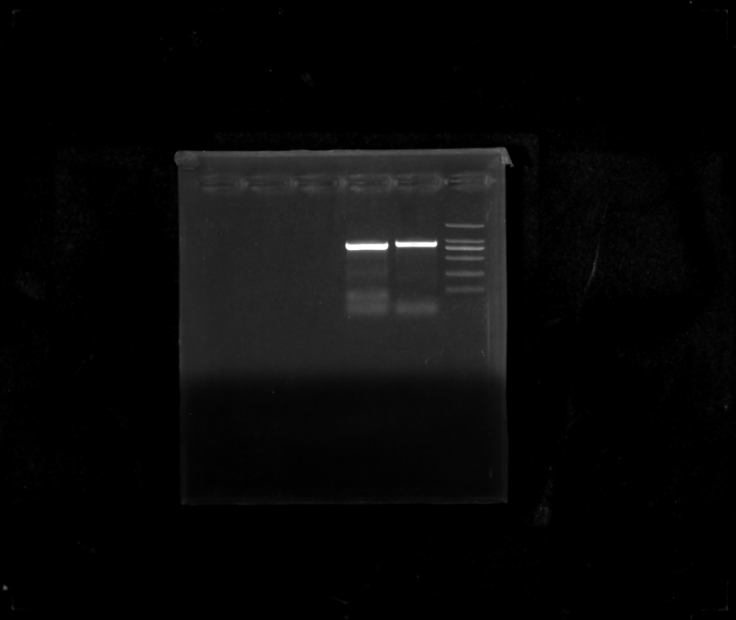


figS2B


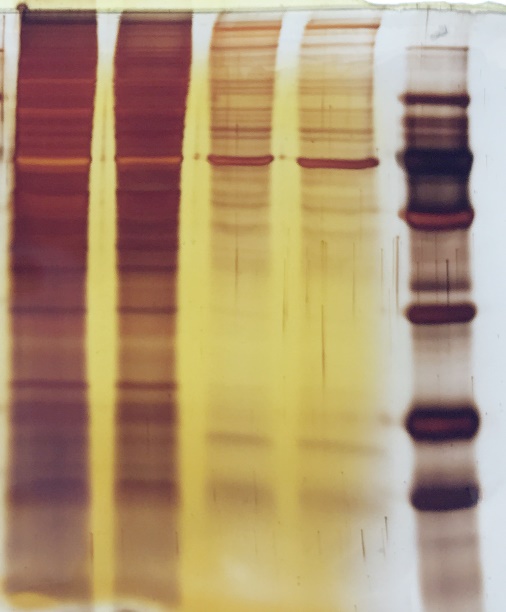

Supplement: Supplementary file 5 — Additional file 5. [file 12864_2022_8923_MOESM5_ESM.docx]
