# Supplementary figures and images for: The long non-coding RNA lncMYOZ2 mediates an AHCY/MYOZ2 axis to promote adipogenic differentiation in porcine preadipocytes
Source: BMC Genomics. 2022 Oct 11;23:700. doi: 10.1186/s12864-022-08923-9 (PMC9552422; doi:10.1186/s12864-022-08923-9)

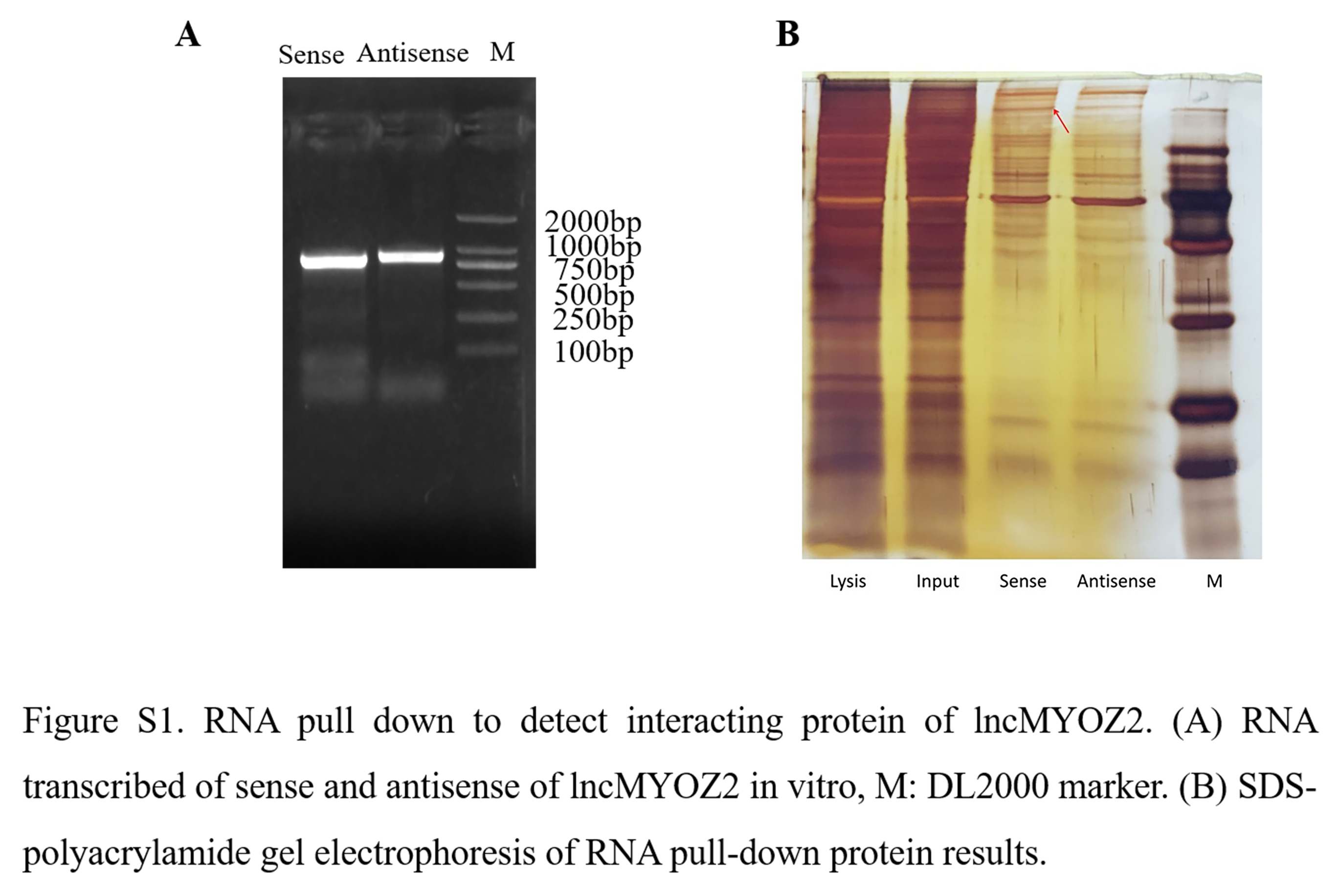

Supplement: Supplementary file 6 — Additional file 6. [file 12864_2022_8923_MOESM6_ESM.tif]
